# Health-related and social factors predicting non-reemployment amongst newly unemployed

**DOI:** 10.1186/1471-2458-12-893

**Published:** 2012-10-23

**Authors:** Mikael Skärlund, Annika Åhs, Ragnar Westerling

**Affiliations:** 1 Department of Public Health and Caring Sciences, Section for Social Medicine, Uppsala University, Box 564, Uppsala 751 22, Sweden

## Abstract

**Background:**

Many researchers have examined the effect that mental health has on reemployment opportunities amongst the unemployed, but the results are inconclusive. Our aim in this study is to investigate the effects that different aspects of mental and physical health, as well as socio-demographic, social, and economic factors, have on reemployment.

**Methods:**

A questionnaire was administered to 1,000 and answered by 502 newly registered unemployed Swedes, who were followed for one year using data from the Swedish National Labour Market Board. The differences between those reemployed and those not reemployed was analysed using stepwise logistic regression.

**Results:**

General mental ill health amongst unemployed individuals measured by the General Health Questionnaire scale was associated with lower levels of reemployment after one year. This effect could not be explained by any of the scales measuring specific aspects of mental disease such as health-related level of function, rate of depression, burnout, or alcohol misuse. Instead being above 45, low control over one’s financial situation, being an immigrant, and visiting a physician during the last three months were better predictors of failure to be reemployed.

**Conclusion:**

There are theoretical reasons to assume that psychological distress leads to a decreased reemployment rate amongst the unemployed. The results of this study partly endorse this hypothesis empirically, showing that general subjective mental distress decreases the rate of reemployment amongst newly unemployed individuals, although this effect was mediated by social and economic factors. Indicators of psychiatric disease had no significant effect on reemployment. The results of this study lead us to suggest the early introduction of financial counselling, psychological support, and other interventions for groups with lower reemployment rates.

## Background

For a large part of the 20^th^ century the high rate of unemployment was an increasingly important concern for most societies. The amount of research on different aspects of employment has increased greatly during the last 30 years. Unemployed individuals suffer from an increase in the release of stress hormones [1-3], a deterioration of health behaviour [4-6], and an increased mortality rate [7-9]. A lot of research has focused on the negative effects unemployment has on mental health [9-12]. It has also been established that mental health is often improved after returning to work [10,11,13-16], and that mental ill health is even seen amongst those made redundant due to plant closures, where personal characteristics are expected to be irrelevant [10,17].

These results support the conclusion that although mental distress may cause unemployment in some cases and keep mentally distressed people out of work in other cases, the most significant correlation between unemployment and mental health is that of causation: unemployment causes mental distress [10,11,18]. Unemployment may cause mental distress through financial deprivation [11,12,19], loss of control [20,21], stress [1], loss of social support [22], and other latent functions associated with employment [23,24].

Fewer studies have focused on the consequences mental ill health has on prospective reemployment opportunities. If mental ill health reduces the possibility of reemployment then this not only supports the hypothesis that there is a selection effect keeping the mentally ill out of work, but it also has wider consequences for unemployment rehabilitation.

Paul and Moser reviewed 49 independent samples in a meta-analysis and concluded that mental ill health actually had a significant negative effect on reemployment possibilities, although the size of the effect was small (effect size = 0,15) [10]. Contrary to this conclusion, another meta-analysis completed by McKee-Ryan et al., which included nine independent samples, could not find any significant correlation between mental distress and reemployment, although the trend was towards a negative effect (effect size = 0,10) [11].

The results regarding the connection between mental health and reemployment are, so far, ambiguous. If unemployed people are to be offered better rehabilitation and support the obstacles for successful reemployment and better health need to be elucidated.

Physical health has been studied less extensively than psychological health, but the general conclusion seems to be that unemployment can deteriorate physical health [11,12,25], although it is disputed by some [26]. Physical health seems to increase the risk of becoming unemployed and may be an impediment to reemployment [25-27].

Reemployment success is also dependent on social factors. Increased efforts are made to find a job when there is a perceived greater financial need, but still a greater financial need leads to a decreased reemployment success [28]. Studies suggest that reemployment may be more difficult for persons from minority groups such as immigrants [29]. Unemployment rate has also been consistently two to three times higher among immigrants than among natives in Sweden [30]. Social support, higher education, lower age, and a higher employment commitment, as well as various personality factors, have been seen in meta-analysis to increase success in a job search and reemployment [28]. a more extensive social network also seems to increase reemployment success [31].

Our aim in this study was to investigate the hypothesis that psychological distress has a negative impact on reemployment and evaluate how different socio-demographic, financial, social, physical, and mental health factors affect opportunities for reemployment.

## Methods

As part of a survey measuring health and healthcare utilisation [32] a questionnaire was sent to 1,000 individuals aged 16–64 who registered themselves as unemployed at the Swedish National Labour Market Board during April 2002. Those included had not been unemployed during 30 days before that date and were still unemployed 25–35 days afterwards. Those who were working but applying for a new job were excluded from the sample. The questionnaire was first received 25–35 days after becoming unemployed, the following dispatches to non-respondents followed during the next weeks.

The questionnaire included information about socio-demographic factors, financial hardship, social support, attitude towards work, contact with a physician during the last three months, and existence of any longstanding illness and depression. The financial section included questions on worrying about personal finances, problems with current expenditure during an ordinary month, if the respondent was able to summon 14,000 Swedish Kronor (approximately €1,600), and if he or she was able to save anything during an ordinary month.

Participants’ health-related level of functioning was estimated using the Swedish version of The Short Form-12 (SF-12) questionnaire, including sub scales on physical (PCS12) and mental (MCS12) health. The answers to the SF-12 were dichotomised using the 25^th^ percentile of the Swedish general population as a cut-off point; participants with higher scores were classified as having a good quality of life [33]. General mental health was estimated using the General Health Questionnaire 12 (GHQ-12) [34] where more than 4 points out of 12 were judged as a cut-off point for having poor general mental health [35]. The Iowa version of the Centre of Epidemiological Studies – Depressed Mood Scale (CES-D) was used to evaluate the existence of depressive symptoms [36,37]. This version sums up to 22 points where higher points indicate more pronounced depressive symptoms. The cut-off point was set at nine, as this was the level achieved by the 80^th^ percentile in a survey sent to the general Swedish population [32]. The existence of mental and physical exhaustion was estimated using the Shirom Melamed Burnout Questionnaire (SMBQ) [38]. In this questionnaire the results were dichotomised according to the results from the general Swedish population, with the 80^th^ percentile, equal to 54 points in an inverted scale, being the cut-off for having a significant burnout [32]. The survey also included The Alcohol Use Disorder Identification Test (AUDIT), which consists of a 0–40 scale where 8 points is the cut-off for an alcohol use disorder [39].

To follow up the employment outcome of the individuals involved, data was obtained from the Swedish National Labour Market Board (AMS) concerning all 1,000 individuals who were sent the survey including information during the 1.5 years after the questionnaire was sent out. The AMS database contains information about new unemployment registrations and the cessation of unemployment registration for every person who has been in contact with an unemployment office, which is necessary in order to obtain unemployment benefits. Information about age, sex, place of residence, and reason for leaving the register was also obtained from the database. To avoid including individuals with only unstable short-term reemployment it was stipulated that for a person to be considered as reemployed he or she had to be employed for at least three months during the following year and to continue being employed one year after first becoming unemployed.

The data was analysed using SPSS 17.0, with a Pearson’s two-sided Chi-2 analysis and logistic regression. The logistic regression analysis was performed with failure to obtain employment as the positive dependent variable. First, bivariate logistic regressions were carried out, thereafter regressions adjusted for sex and age. Finally, a stepwise multivariate logistic regression in three steps was performed containing different models. Model 1 was adjusted for socio-demographic variables: “age”, “sex”, “country of birth”, “place of residence” and “education” as well as “previously unemployment”, as this was known to be a factor associated with not responding. Model 2 also included social factors: “emotional support”, “social network size” and “civil status” as well as “monthly savings”, which was the only economic factor with significant outcome in the bivariate analysis. Model 3 further added health factors that were significant in the bivariate model: “visits to a physician” and “GHQ-12”.

The questionnaire had a response rate of 56.9% after four waves of reminders; the last respondents answering three months after becoming registered as unemployed. A total of 128 individuals (12.8%) had been out of contact with the AMS register for unknown reasons for more than three months during the year and thus could not be included in the study; half of these, 6.1%, were both non-respondents from the questionnaire and had information missing from the register (see Table 1). This resulted in 50.2% (*n* = 502) of the sample being included in the study and 49.8% (*n* = 498) being excluded from the study mostly due to low response rate to the questionnaire. Thanks to the dual sources of information some data about the persons excluded could be obtained. To estimate non-response bias a logistic regression analysis was performed with exclusion from the study as the dependent variable. It resulted that age below 45, male sex, living in a mayor city and earlier unemployment experience were all independent factors correlated to a higher rate of exclusion from the study (Table 1). There was no significant exclusion bias registered among the variables taken from the questionnaire (results not shown).

**Table 1:** Proportion and logistic regression of non-response by sociodemographic factors

		**Included**	**Excluded**	**Logistic regression**
**Total**		502 (50.2%)	498 (49.8%)	
**Age**	17-25	178 (47.8%)**	194 (52.2%)**	1
	26-45	231 (47.8%)**	252 (52.2)**	0.89 (0.65-1.23)
	46-64	93 (64.1%)**	52 (35.9%)**	0.53 (0.34-0.82)**
**Sex**	Female	233 (58.7%)**	164 (41.3%)**	1
	Male	269 (44.6%)**	334 (55.4%)**	1.84 (1.38-2.45)**
**Place of residence**^**a**^	Mayor city	150 (42.3%)**	205 (57.7%)**	**1**
	Other cities	147 (53.6%)**	127 (46.4%)**	0.66 (0.46-0.94)*
	Smaller cities and rural districts	205 (55.3%)**	166 (44.7%)**	0.62 (0.45-0.86)**
**Previous unemployment**	No	284 (54.3%)**	239 (45.7%)**	**1**
	Yes	218 (45.7%)**	259 (54.3%)**	1.67 (1.25-2.24)**
**Reemployment**^**b**^	Reemployed	81 (64.3%)	45 (35.7%)	1
	Not reemployed	421 (56.4%)	325 (43.6%)	1.43 (0.95-2.14)

* = p < 0.05 ** = *p* < .01 Pearson’s two-sided Chi-2 significance.

^a^ = Mayor city = district with > 200,000 inhabitants plus suburban districts adjacent to these districts. Other city = district with 50,000-200,000 inhabitants. In accordance with Svenska Kommunförbundet [56].

^b^ = Had been employed at least three months and had employment after one year. Concerns only those 872 with complete data from the AMS register.

^L^ = Multivariate logistic regression with exclusion as the dependent variable and age, sex, place of residence, previous unemployment and non-reemployment as independent variables.

To account for the non-response bias the variables with data not missing at random (“age group”, “sex”, “place of residence” and “former unemployment”) were post stratified by the model presented by Carlin et al. [40]. All the analyses were then re performed once again while weighting for the non-response bias. None of the result values nor the significances were changed in any major sense. Results for the weighted version of model 3 in the logistic regression analysis is included in Table 2.

**Table 2:** Logistic regression of factors predicting non-reemployment

**OR for failure of reemployment (95% confidence interval)**						
	**Unadjusted**	**Adjusted for age and sex**	**Model 1**	**Model 2**	**Model 3**	**Model 3 weighted**
**Age**						
16-25	**1**	**1**	**1**	**1**	**1**	**1**
26-45	1.20 (0.72-2.00)	1.21 (0.72-2.01)	1.05 (0.59-1.87)	1.18 (0.64-2.17)	1.25 (0.67-2.32)	1.26 (0.68-2.35)
46-65	2.20 (1.01-4.82)*	2.28 (1.04-4.99)*	2.09 (0.90-4.84)	2.70 (1.09-6.70)*	2.96 (1.12-7.80)*	3.16 (1.09-9.14)*
**Being a man**	0.81 (0.50-1.30)	0.77 (0.48-1.25)	0.75 (0.45-1.23)	0.69 (0.41-1.16)	0.76 (0.44-1.31)	0.77 (0.44-1.35)
**Born outside Sweden**	2.26 (1.09-4.70)*	2.23 (1.07-4.67)*	2.38 (1.11-5.11)*	2.09 (0.95-4.61)	1.83 (0.79-4.26)	1.79 (0.79-4.05)
**Education**						
University (reference)	1	1	1	1	1	1
Secondary school	0.93 (0.51-1.67)	1.08 (0.58-2.01)	1.16 (0.61-2.20)	1.09 (0.56-2.12)	1.04 (0.53-2.08)	0.95 (0.47-1.89)
Lower secondary school	1.33 (0.60-2.94)	1.26 (0.55-2.89)	1.29 (0.55-3.03)	1.14 (0.47-2.76)	1.02 (0.40-2.59)	0.83 (0.32-2.11)
Other	0.83 (0.36-1.91)	0.81 (0.35-1.90)	0.79 (0.33-1.89)	0.64 (0.26-1.58)	0.58 (0.23-1.51)	0.47 (0.18-1.19)
**Place of residence**^**b**^						
Mayor city reference)	1	1	1	1	1	1
Other cities	1.08 (0.58-2.03)	1.05 (0.56-1.98)	1.1 (0.61-2.25)	1.24 (0.64-2.21)	1.16 (0.59-2.30)	1.15 (0.59-2.23)
Smaller cities and rural districts	0.93 (0.52-1.63)	0.89 (0.50-1.58)	0.96 (0.51-1.78)	1.10 (0.58-2.10)	1.17 (0.60-2.27)	1.23 (0.64-2.36)
**Being married/cohabiting**	0.91 (0.56-1.47)	0.73 (0.44-1.24)		0.70 (0.39-1.22)	0.67 (0.38–1.19)	0.64 (0.35-1.14)
**A big social network**^**c**^	0.80 (0.58–1.11)	0.80 (0.57–1.11)		0.93 (0.65–1.32)	0.97 (0.67–1.40)	1.00 (0.71-1.44)
**High emotional support**	0.66 (0.36–1.20)	0.64 (0.35–1.19)		0.79 (0.41–1.53)	0.79 (0.38–1.64)	0.83 (0.41-1.69)
**Not being able to do monthly savings**	1.91 (1.18–3.11)**	2.03 (1.24–3.32)**		1.74 (1.03–2.91)*	1.77 (1.03–3.04)*	1.83 (1.06-4.14)*
**Not managing to summon 14 000 SEK**	1.38 (0.82–2.33)	1.46 (0.86–2.47)				
**Having difficulties with expenditures**	1.44 (0.88–2.37)	1.56 (0.94–2.59)				
**Being worried over economy**	1.25 (0.71–2.21)1.35 (0.76–2.42)	1.25 (0.71–2.21)1.35 (0.76–2.42)				
**Considering work important**	0.76 (0.40–1.45)0.77 (0.40–1.47)	0.76 (0.40–1.45)0.77 (0.40–1.47)				
**Previously unemployed**	1.46 (0.89–2.39)	1.45 (0.87–2.43)	1.63 (0.96-2.78)		1.31 (0.75-2.39)	1.32 (0.76-2.29)
**Bad self-rated health**	1.86 (0.86–4.03)	1.71 (0.78–3.74)				
**Longstanding illness**	1.65 (0.85–3.19)	1.65 (0.85–3.19)				
**Visit to a physician last 3 months**	1.96 (1.18–3.23)**	1.80 (1.07–3.01)*			1.61 (0.94–2.27)	1.51 (0.88-2.60)
**Bad physical functioning level (PSC**^**d**^**)**	1.51 (0.82–2.78)	1.39 (0.75–2.58)				
**General mental distress (GHQ-12**^**e**^**)**	1.86 (1.13–3.05)*	1.94 (1.17–3.22)*			1.39 (0.79–2.45)	1.38 (0.78-2.44)
**Bad mental functioning level (MCS**^**f**^**)**	1.26 (0.77–2.07)	1.35 (0.81–2.23)				
**Depression (subjective)**	2.60 (0.78–8.64)	2.66 (0.80–8.88)				
**Depression according to CES-D**^**g**^	1.05 (0.58–1.90)	1.09 (0.60–1.99)				
**Being burned out (SMBQ**^**h**^**)**	1.20 (0.66–2.18)	1.21 (0.66–2.22)				
**Risky alcohol use (AUDIT**^**i**^**)**	0.76 (0.44–1.32)	0.92 (0.51–1.64)				

A higher value indicates an increased odds ratio for not obtaining new employment.

Model 1: Adjusted for age, sex, education, country of birth, place of residence and earlier unemployment.

Model 2: Adjusted for model 1 + monthly savings, emotional support, size of social network and civil status.

Model 3: Model 2 + GHQ-12 and visits to a physician.

Model 3 weighted: Model 3 while weighting for non-response bias according to the model used by Carlin et al. [40].

* = p < .05. ** = p < .01.

b = Mayor city = district with > 200,000 inhabitants plus suburban districts adjacent to these districts. Other city = district with 50,000-200,000 inhabitants. In accordance with Svenska Kommunförbundet [56].

c = How many individuals do you know and have contact with and with whom you share the same interests?

d = SF-12 Physical Component Study.

e = General Health Questionnaire-12.

f = SF-12 Mental Component Study. g = Centre of Epidemiological Studies - Depression scale.

h = Shirom Melamed Burnout Questionnaire.

I = Alcohol Use Disorders Identification Test (AUDIT).

This study was approved by the ethics committee of Uppsala University, Dnr 02–156. It was carried out in cooperation with the Swedish National Labour Market Board and all participation was voluntary.

## Results

The majority of the total sample were young (median age = 30), 53.6% male, 20.6% born outside Sweden, 29.9% lived in a major city, and 40.8% in municipalities with less than 50,000 inhabitants; 25.3% were university graduates, and the highest level of education for 46.2% of them was secondary school. Only 42.9% were able to save on a monthly basis. A total of 50.4% of the sample had recently visited a physician, and 22.2% considered themselves to have a longstanding illness, 50.6% suffered from subjective mental distress according to GHQ-12, but only 8.3% considered themselves to be depressed on a direct question (see Table 3).

**Table 3:** Distribution of participants by sociodemographic factors, social and economic situation, and health

		**Frequency**	**Percentage**			**Frequency**	**Percentage**
**Reemployment**^**a**^	Reemployed	81	16.1%	**Worried over economy**	No	100	20.6%
	Not reemployed	421	83.9%		Yes	386	79.4%
**Age**	17-25	178	35.5%	**Significance of work**	Not important	96	20.1%
	26-45	231	46.0%		Important	381	79.9%
	46-64	93	18.5%	**previously unemployment**	No	284	56.6%
**Sex**	Female	233	46.4%		Yes	218	43.4%
	Male	269	53.6%	**Longstanding illness**	No	376	77.8%
**Place of birth**	Sweden	392	79.5%		Yes	107	22.2%
	Abroad	101	20.5%	**Visit to a physician**	No	239	50.4%
**Education**	University	124	25.3%		Yes	235	49.6%
	Secondary school	227	46.2%	**Self-rated health**	Good	411	84.0%
	Lower secondary school	87	17.7%		Bad	78	16.0%
	Other	53	10.8%	**PSC**^**d**^	Better functioning	335	74.8%
**Civil status**	Single	231	47.0%		Worse functioning	113	25.2%
	Married/Cohabiting	261	53.0%	**GHQ-12**^**e**^	No mental distress	233	49.4%
**Place of residence**^**b**^	Mayor city	150	29.9%		Mental distress	239	50.6%
	Other cities	147	29.3%	**MSC**^**f**^	Better functioning	237	52.9%
	Smaller cities and rural districts	205	40.8%		Worse functioning	211	47.1%
**Social network size**^**c**^	Low	100	20.6%	**Depression**	No depression	452	91.7%
	High	386	79.4%		Depression	41	8.3%
**Emotional support**	Low	120	24.8%	**CES-D**^**g**^	Low degree of dep.	356	76.9%
	High	363	75.2%		High degree of dep.	107	23.1%
**Monthly saving**	Yes	205	42.9%	**Burnout**^**h**^	Low burnout	337	75.4%
	No	273	57.1%		High burnout	110	24.6%
**Manage sudden expend**							
	Yes	305	63.5%	**Risky alcohol use**^**i**^	No risky alcohol use	343	75.4%
	No	175	36.5%		Risky alcohol use	112	24.6%
**Difficulties with current expenditures**	No	273	56.4%				
	Yes	211	43.6%				

^a^ = Had been employed at least three months and had employment as last registration.

^b^ = Mayor city = district with > 200,000 inhabitants plus suburban districts adjacent to these districts. Other city = district with 50,000-200,000 inhabitants. In accordance with Svenska Kommunförbundet [56].

^c^ = How many individuals do you know and have contact with and with whom you share the same interests?

^d^ = Physical functioning level according to SF-12 Physical Component Study.

^e^ = General Health Questionnaire-12.

^f^ = Mental functioning level according to SF-12 Mental Component Study.

^g^ = Centre of Epidemiological Studies - Depression scale.

^h^ = Shirom Melamed Burnout Questionnaire.

^I^ = Alcohol Use Disorders Identification Test (AUDIT).

After one year 16.1% (*n* = 81) had obtained stable employment. reemployment was less common among those who were born outside Sweden, had visited a doctor more often during the last three months, had a longstanding illness, had a worse score on the GHQ-12 scale, and were less able to make monthly savings (see Table 4).

**Table 4:** Proportions of participants reemployed by sociodemographic factors, social and economic situation, and health

		**Reemployed**^**a**^		**Not reemployed**		**Sig.**^†^		**Reemployed**^**a**^			**Not reemployed**		**Sig.**^†^
**Age**	17-25	34	42,0%	144	34,2%		**Worried over economy**	No	19	23,8%	81	20,0%	
	26-45	38	46,9%	193	45,8%			Yes	61	76,3%	325	80,0%	
	46-64	9	11,1%	84	20,0%		**Importance of work**	Not important	65	83,3%	316	79,2%	
**Sex**	Female	34	42,0%	199	47,3%			Important	13	16,7%	83	20,8%	
	Male	47	58,0%	222	52,7%		**Previous unemployment**	No	52	64,2%	232	55,1%	
**Place of birth**	Sweden	71	88,8%	321	77,7%	*		Yes	29	35,8%	189	44,9%	
	Abroad	9	11,3%	92	22,3%	*	**Longstanding illness**	No	68	85,0%	308	76,4%	
**Education**	University	20	25,0%	104	25,3%			Yes	12	15,0%	95	23,6%	
	Secondary school	39	48,8%	188	45,7%		**Visit to physician**	No	50	64,1%	189	47,7%	**
	Lower secondary school	11	13,8%	76	18,5%			Yes	28	35,9%	207	52,3%	**
	Other	10	12,5%	43	10,5%		**Self-rated health**	Good	72	90,0%	339	82,9%	
**Civil status**	Single	36	45,0%	195	47,3%			Bad	8	10,0%	70	17,1%	
	Married/Cohabiting	44	55,0%	217	52,7%		**PSC**^**d**^	Better functioning	63	80,8%	272	73,5%	
**Place of residence**^**b**^	Mayor city	24	29,6%	126	29,9%			Worse functioning	15	19,2%	98	26,5%	
	Other cities	22	27,2%	125	29,7%		**GHQ-12**^**e**^	No mental distress	49	62,0%	184	46,8%	*
	Smaller cities and rural districts	35	43,2%	170	40,4%			Mental distress	30	38,0%	209	53,2%	*
**Social network**^**c**^	Low	12	15,0%	88	21,7%		**MSC**^**f**^	Better functioning	45	57,7%	192	51,9%	
	High	68	85,0%	318	78,3%			Worse functioning	33	42,3%	178	48,1%	
**Emotional support**	Low	15	18,8%	105	26,1%		**Depression**	No depression	77	96,3%	375	90,8%	
	High	65	81,3%	298	73,9%			Depression	3	3,8%	38	9,2%	
**Monthly savings**	Yes	45	56,3%	160	40,2%	**	**CES-D**^**g**^	Low degree of depression	56	76,7%	297	76,7%	
	No	35	43,8%	238	59,8%	**		High degree of depression	17	23,3%	90	23,3%	
**Manage sudden expenditure of 14,000**	Yes	55	69,6%	250	62,3%		**Burnout**^**h**^	Low burnout	57	78,1%	280	74,9%	
	No	24	30,4%	151	37,7%			High burnout	16	21,9%	94	25,1%	
**Difficulties with current expenditures**	No	51	63,8%	222	55,0%		**Risky alcohol use**^**i**^	No risky alcohol use	54	71,1%	289	76,3%	
	Yes	29	36,3%	182	45,0%			Risky alcohol use	22	28,9%	90	23,7%	

a = Had been employed at least three months and had employment as last registration.

b = Mayor city = district with > 200,000 inhabitants plus suburban districts adjacent to these districts. Other city = district with 50,000-200,000 inhabitants. In accordance with Svenska Kommunförbundet [56].

c = How many individuals do you know and have contact with and with whom you share the same interests?

d = Physical functioning level according to SF-12 Physical Component Study.

e = General Health Questionnaire-12.

f = Mental functioning level according to SF-12 Mental Component Study.

g = Centre of Epidemiological Studies - Depression scale.

h = Shirom Melamed Burnout Questionnaire.

I = Alcohol Use Disorders Identification Test (AUDIT).

† = Chi-2-square significance. * = p < .05. ** = p < .01.

### Multivariate analyses

Having visited a physician during the last three months and having a worse level of subjective mental health measured by GHQ-12 were both significant factors predicting non-reemployment when adjusted for age and sex, but their significance disappeared when social and economic factors were included. Other measurements of health, including self-rated health, longstanding illness, PCS12, MCS12, depression, CES-D, SMBQ, and AUDIT, were not significantly related to non-reemployment (Table 2). As “GHQ-12” and “visits to a physician” could be expected to be correlated an alternative Model 3 including only one of these factors at the time plus social- and economic factors was tested, without significance for any of the two factors.

Amongst the socio-demographic factors in the regression analysis, being older than 45 and being born outside Sweden had a significant negative effect on reemployment outcome when adjusting for basic socio-demographic factors. The effect of being an immigrant disappeared when social and economic factors were included in the analysis. The effect of age seemed partly due to other socio-demographic factors as the significance was lost when those other factors were included, but the effect was reinforced and was significant when economic, social, and health factors were included (Odds Ratio *(OR)* = 2.96). Sex, education, and place of residence did not have any significant effect on reemployment outcome.

Not being able to save on a monthly basis was a significant predictor leading to a decreased level of reemployment even when adjusted for health, socio-demographic, and social factors (*OR* = 1.77), while the other economic questions were without significance. Social factors such as civil status, emotional support, social network size, previous unemployment experiences, and considering work important did not have any significant influence over the rate of reemployment.

## Discussion

Older individuals, immigrants, and individuals with a narrower financial margin had greater difficulty finding a new job. The same was true for individuals with subjective mental distress measured by GHQ-12 and individuals who had recently had contact with a physician, although these effects seemed to be partially mediated by socio-demographic- and financial factors.

### Health factors

Having a poor level of general subjective mental health (measured by GHQ-12) was a significant predictor of failing to be reemployed when adjusted for sex and age. However the factor of psychological distress was not independent of social and economical factors. The analysis did not show any significant results regarding measurements of psychiatric diseases. Although not significant, the direction of the correlations between the measurements of psychiatric disease and reemployment were those expected, with more indicators of psychiatric disease being associated with somewhat lower chances of reemployment. This study therefore gives some support to earlier studies connecting a poor self-rated mental health, measured by GHQ-12, with lower chances of reemployment [13,21,41,42]. It also provides support for studies that provide evidence that there is no correlation between indicators of psychiatric disease and reemployment using Symptoms Checklist 90 [15,43], Hopkins Symptoms Checklist (HSC) [44], Brief Symptom Inventory for depression [41] or CES-D [16]. There are, however, several other researchers using HSC, who arrived at a different conclusion [17,42,45]. In general, however, in both the studies of self rated mental health and in the three studies finding a significant effect of psychiatric symptoms, the effect sizes on reemployment were small. There may be reasons to think that subjective mental health is a better predictor of reemployment than psychiatric disease. Psychiatric disease usually leads to medical contact, treatment, and sick leave. However, subjective mental distress may become a chronic daily impediment more often without either cure or support. It should be stressed, though, that we do not know whether or not the distress detected amongst the newly unemployed in this sample existed before the individuals became unemployed.

There may be several reasons for an association between psychological distress and decreased reemployment. Psychological distress might decrease the unemployed person’s efforts to find a job [21], which decreases chances of reemployment [28]. Mental ill health may inhibit unemployed persons from participating in active labour market programs facilitating reemployment. Employers may also be disinclined to engage an unemployed person with psychological distress, expecting him or her to do an inferior job. Failure of reemployment and psychological distress may as well be correlated because of a common underlying cause, such as personality. Low level of extroversion, agreeableness, conscientiousness, and openness have been correlated both to decreased subjective well-being in general [46] and lower prospects for acquiring new employment amongst the unemployed [28]. Some personality features may also impair coping with unemployment. Mental ill health might also be the rational response of a person who rightly expects to have a hard time getting new employment, while individuals accurately anticipating being swiftly reemployed may consider a spell of unemployment to be a less negative experience.

Considering the role of physical health amongst the newly unemployed, it is unclear why individuals who have recently visited a physician have a hard time finding a new job when having a longstanding illness, a poor physical functioning level (measured using the PCS), or bad self-rated health do not have any significant correlation with reemployment. The trend, however, is towards a negative effect. It might be that visits to a physician, without having a longstanding disease, reflect subjective mental distress here. However, it has been reported elsewhere that poor physical health can be an impediment to reemployment [27].

### Economic factors

This study shows that individuals unable to save money during a normal month had lower rates of reemployment. However, being worried about personal finance, having problems with one’s current expenditure, or not being able to summon a large amount of money within one week did not have a significant effect. This study was performed in a Swedish setting where income poverty is not considered a major problem during unemployment, as most people are members of unemployment insurance funds. Those who do not receive unemployment compensation from a fund are normally supported by social allowances. Yet it remains the case in Sweden that unemployed people live under financial strain three times as often as employed people [32]. Studies have shown that higher levels of unemployment compensation reduce levels of reemployment [47,48]. However, other studies on reemployment have concluded that better personal finance is associated with higher levels of reemployment [28,45]. It has also been shown that unemployment benefits have a positive effect on the mental health of the unemployed [47,49]. The financial variable measured in this study is the ability to save during a normal month, which may indicate a person’s control over his or her finances rather than the general economic situation. It might also be that individuals with a better financial situation also possess other personal resources that make them more employable. As expected, and in accordance with official statistics, those unemployed born outside Sweden and older individuals had lower rates of reemployment . Both these difficulties may be at least partly an effect of discrimination [50,51]. More surprising is the lack of influence exerted by education, place of residence, social network, and social support.

### Methodological issues

Regarding methodological issues, it should be noted that the reemployment figures were drawn from a register that recorded different reasons for ceasing to be unemployed, but which did not have a special category for sick leave. Instead individuals who ceased to be registered unemployed because they were given sick leave were included with those who had other reasons for ceasing to be registered unemployed. Excluding the two small categories that may include individuals leaving the workforce due to ill health from the analysis did not change the results in any significant respect (results not shown). This study also had a high level of non-respondents (49.8%), and some groups of unemployed people seem to be under-represented in the study, especially males, individuals living in major cities and those previously unemployed. However the regression models were adjusted for these factors and weighted in order to lessen the influence of the non-response bias on the results. There were no major differences in the results after this adjustment. Non-respondents to the questionnaire (43.1%) did later become reemployed to a somewhat lower degree than respondents, although the difference was not significant. Meanwhile those 12.8% excluded because of missing information from AMS could according to earlier studies be expected to have become reemployed to a higher degree. According to previous investigations 39% of the individuals leaving the AMS register for unknown reasons did so because they had obtained new employment without informing the authority [52]. These effects may have biased the study, though it is unclear in which direction. Multivariate and bivariate logistic regression analyses were also performed including only late respondents to the questionnaire, including persons responding after the third or fourth reminder. The analyses revealed a very similar pattern to that presented above. For some of the questions in the questionnaire, for instance the direct question: “Do you have a depression?” the positive answers were too small to draw any distinct conclusions on significant effects. This does not explain the differences found in effects between indicators of psychiatric disease and mental distress, where positive answers to the questionnaires exceeded 100. It should also be noted that this study is not representative of the entire unemployed population in Sweden, as the sample was drawn from individuals who had been unemployed for a short period (25–35 days) and at a particular time of year. Therefore, no definite implications regarding the effect that mental health has on reemployment amongst the long-term unemployed can be drawn. Even so, the study suggests several implications for policy makers.

### Implications

The negative effect of psychological distress and financial control on reemployment leads to the suggestion that psychological support and treatment as well as financial counselling can be important measures in the active labour market policy early in the unemployment process. Interventions based on cognitive-behavioural therapy have shown positive effects on reemployment amongst long-term unemployed [53] and may also have an effect even early in the unemployment process. An intervention in Michigan focusing on acquiring job search, problem-solving skills, and preparedness against setbacks managed to both increase reemployment and decrease psychological distress amongst unemployed [45], though similar approaches elsewhere have failed to show any of these effects [54]. Further studies are necessary to reveal how these labour market policies should be designed. To alleviate the situation for unemployed immigrants an active policy against discrimination is essential as well as research-based labour market policies directed at training and support with job searching for affected individuals [55].

## Conclusion

This study partly supports the hypothesis that psychological distress amongst the newly unemployed obstructs reemployment, though no specific psychiatric disease could explain this effect. The study further introduces the importance of financial control, age, and immigration status for reemployment success: individuals with poor financial control, those older than 45, and immigrants had a harder time finding new employment. The results suggest that measures should be put in place to counteract these differences through financial counselling, psychological treatment, and active labour market policies. Future studies should investigate different aspects of psychological distress and, instead of treating mental disease and mental health as a single concept, try to elucidate which aspects really affect reemployment and how the interventions that aim to facilitate reemployment should be designed.

## Competing interests

The authors declare that they have no competing interests.

## Authors' contributions

MS participated in the data analysis and in writing the article. AÅ designed and carried out the questionnaire and collected the data. RW participated in designing the questionnaire, interpreting the data, and revising the article. All authors read and approved the final manuscript.

## Pre-publication history

The pre-publication history for this paper can be accessed here:

http://www.biomedcentral.com/1471-2458/12/893/prepub
